# Modular Platform for Efficient Assembly of Multifunctional Antibodies Using Orthogonal Protein–Protein Interactions

**DOI:** 10.1021/acsami.4c21958

**Published:** 2025-03-31

**Authors:** Baizhen Gao, Rushant Sabnis, Siddhi Kotnis, Sofia Feliciano, Kyge Poling, Tracy Mei, Min Feng, Jugal Kishore Das, Jianxun Song, Qing Sun

**Affiliations:** † Department of Chemical Engineering, Texas A&M University, College Station, Texas 77840, United States; ‡ Department of Microbial Pathogenesis and Immunology, Texas A&M University Health Science Center, Bryan, Texas 77807, United States; § Interdisciplinary Graduate Program in Genetics and Genomics, Texas A&M University, College Station, Texas 77843, United States

**Keywords:** antibody engineering, protein−protein interaction, protein purification, nanobody, elastin-like polypeptide (ELP)

## Abstract

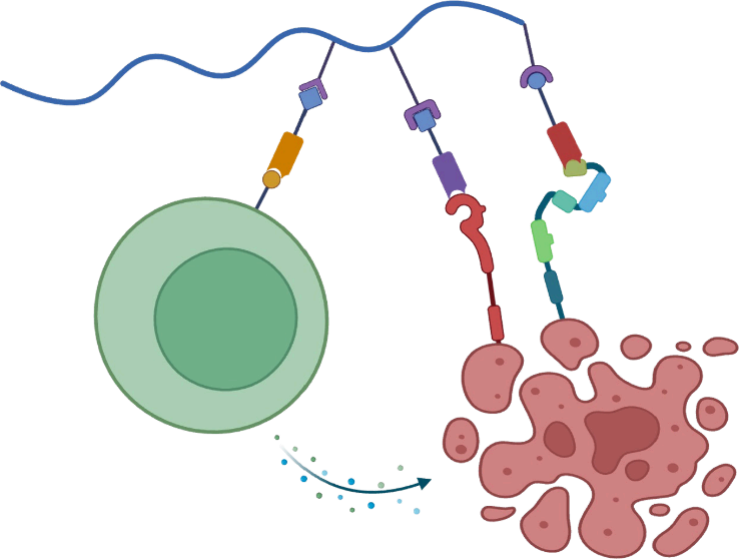

Multifunctional antibodies, capable of simultaneously engaging multiple targets, are a unique class of antibodies that have sparked growing interest. Current approaches for making multifunctional antibodies, including chemical conjugation or genetic modifications, suffer from low product yield, complex structure design, and complicated manufacturing processes. In this study, we report a modular post-translational platform with highly specific protein–protein interactions for multifunctional antibody assembly and an elastin-like polypeptide (ELP) for easy purification. We generated and purified multifunctional antibodies with over 90% assembled scaffold and overall product purity. Additionally, we assembled antibodies with diverse applications, including detecting cancer, inhibiting cancer cell growth, and directing T cells to cancer cells for enhanced therapeutic efficacy. This platform offers high assembly efficiency, easy purification, and modularity for the redesign of antibody functions.

## Introduction

Multifunctional antibodies, equipped with multiple binding motifs, are a unique class of antibodies. They exhibit enhanced binding affinity and specificity, which lower the risk for on-target, off-tumor specificities. Multifunctional antibodies can direct specific immune cells to tumors. The Federal Drug Agency (FDA) and European Medicine Agency (EMA) have granted approval for blinatumomab, a bispecific T-cell engager that simultaneously attaches to T cells and cancer cells, enabling T cells to find and destroy cancer cells by bringing them close together. Blinatumomab has been shown to be more effective than chemotherapy in treating children and young adults with B-cell acute lymphoblastic leukemia that has come back after initial treatment. By targeting multiple antigens on a single target cell, multifunctional antibodies may prevent antigen-negative relapses. This is achieved by exerting therapeutic pressure through either sequential or simultaneous targeting, which helps inhibit the development of negative variants.^1^

Although promising, the intricate structures of multifunctional antibodies pose a significant challenge in their production.^2,3^ Current strategies, including IgG-like or non-IgG-like multifunctional antibody manufacturing, using genetic engineering and chemical modification, suffer from reduced yield, increased purification complexity, and restricted spatial geometric arrangements of domains.^2,3^

In this work, we develop a protein scaffold approach for efficiently assembling multifunctional antibodies utilizing three highly specific orthogonal protein interaction pairs. One of the widely used protein–protein interactions is the SpyTag-SpyCatcher interaction. Originated from the bacterium *Streptococcus pyogenes* fibronectin-binding protein FbaB, SpyTag and SpyCatcher spontaneously and irreversibly form a covalent bond between lysine on SpyCatcher and aspartic acid on SpyTag when mixed.^4^ This reaction can tolerate a wide range of pH and temperature conditions, and both SpyTag and SpyCatcher can be fused to either the N- or C-terminal of the protein of interest.^4^ Following the initial engineering of the SpyTag/SpyCatcher pair, SpyTag002/SpyCatcher002 was developed through phase-display library screening, leading to an order of magnitude enhancement in interaction kinetics.^5^ Besides faster kinetics, the orthogonal interaction pair SnoopTag/SnoopCatcher was also developed to form a covalent bond between lysine on SnoopTag and asparagine on SnoopCatcher by engineering the adhesin RrgA from *Streptococcus pneumoniae.*(6) We use SpyTag002/SpyCatcher002 and SnoopTag/SnoopCatcher protein pairs to facilitate the spontaneous covalent attachment between protein partners.^4,6,7^ Besides covalent bonding, cohesin–dockerin represents one of the strongest protein–protein interaction pairs.^8^ Dockerin binds with cohesin through a duplicated 22-residue calcium binding loop–helix F-hand motif.^8^ Because of its strong interaction, we also used the cohesin–dockerin pair to assemble a third antibody fragment to further enhance the assembled antibody capability. These systems have been previously used to assemble antibodies and other *in vivo* applications.^9−19^ We further fuse SpyCatcher002, SnoopCatcher, and dockerin with biocompatible elastin-like polypeptide (ELP),^20^ which allows easy separation of the target protein from host cell lysis through thermal precipitation cycles (Figure S1).

**Figure 1:**
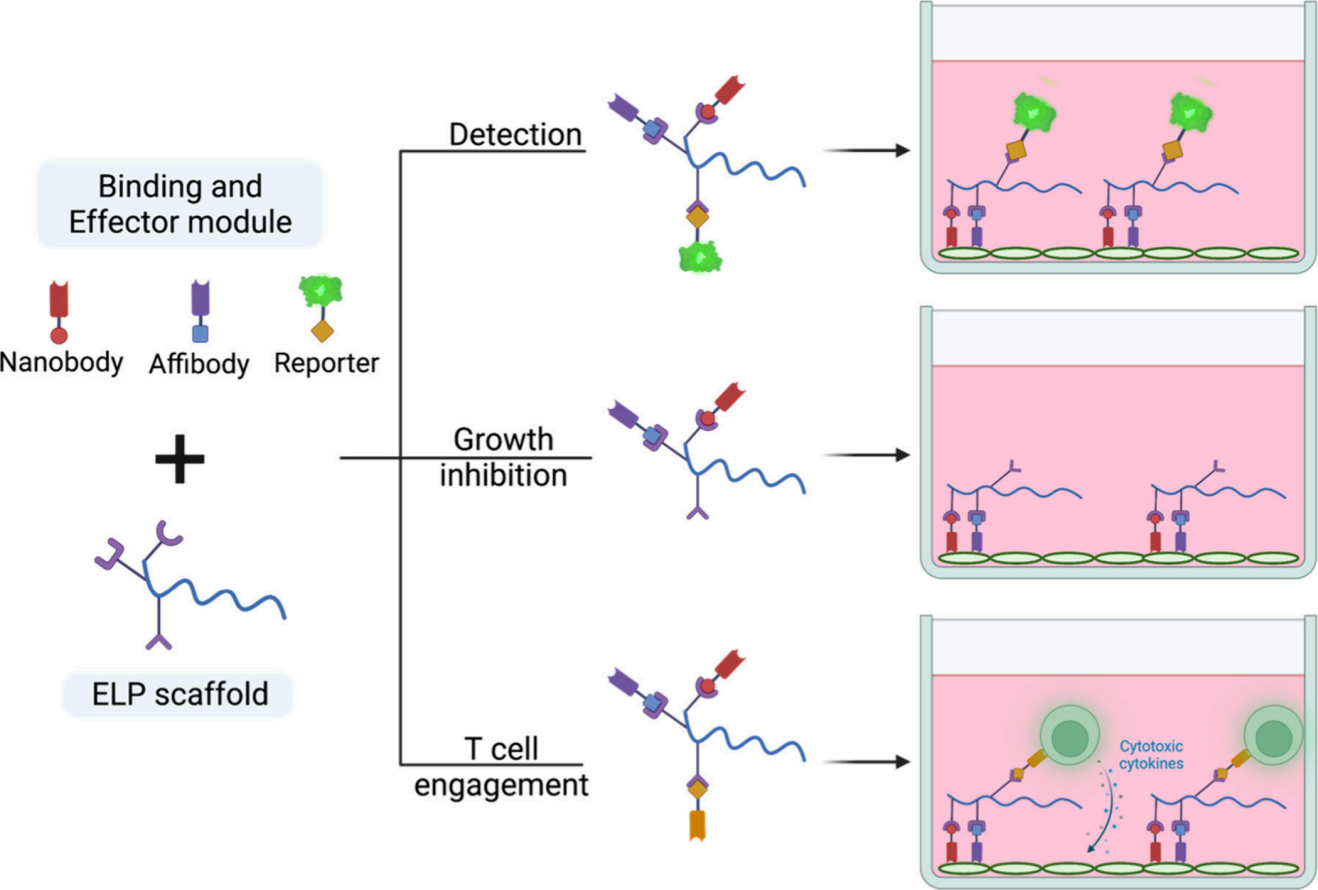
Schematic illustration of post-translational assembly of a multifunctional antibody.

We assemble our multifunctional antibody using nanobodies and affibodies. Nanobodies and affibodies are small biomolecules derived from the heavy chain of the Camelidae family and *staphylococcal* protein A, respectively.^21,22^ They present an appealing solution for targeting cancer biomarkers due to their low immunogenicity and high targeting specificity.^23^ This protein-scaffold-based strategy leads to over 90% of the assembled scaffold by utilizing extremely precise protein–protein interactions. We counteract the purification challenge faced by multifunctional antibodies through the ELP purification tag. Furthermore, we demonstrate the modularity of multifunctional antibodies by detecting cancer, inhibiting cancer cell growth, and directing T cells to cancer cells for high therapeutic efficacy (Figure 1).

## Results

### Antibody Assembly

For our antibody assembly platform, we started with HER2 and EGFR as the targeting cancer biomarkers, given their documented overexpression in various tumor types, including breast and lung cancer.^24,25^ Both HER2 and EGFR are part of the epidermal growth factor receptor family, and they are responsible for activation of the downstream signaling pathways that regulate cell cycle progression.^26,27^ Previous studies have shown that simultaneous inhibition of both HER2 and EGFR significantly enhances therapeutic efficacy.^28^

**Figure 2:**
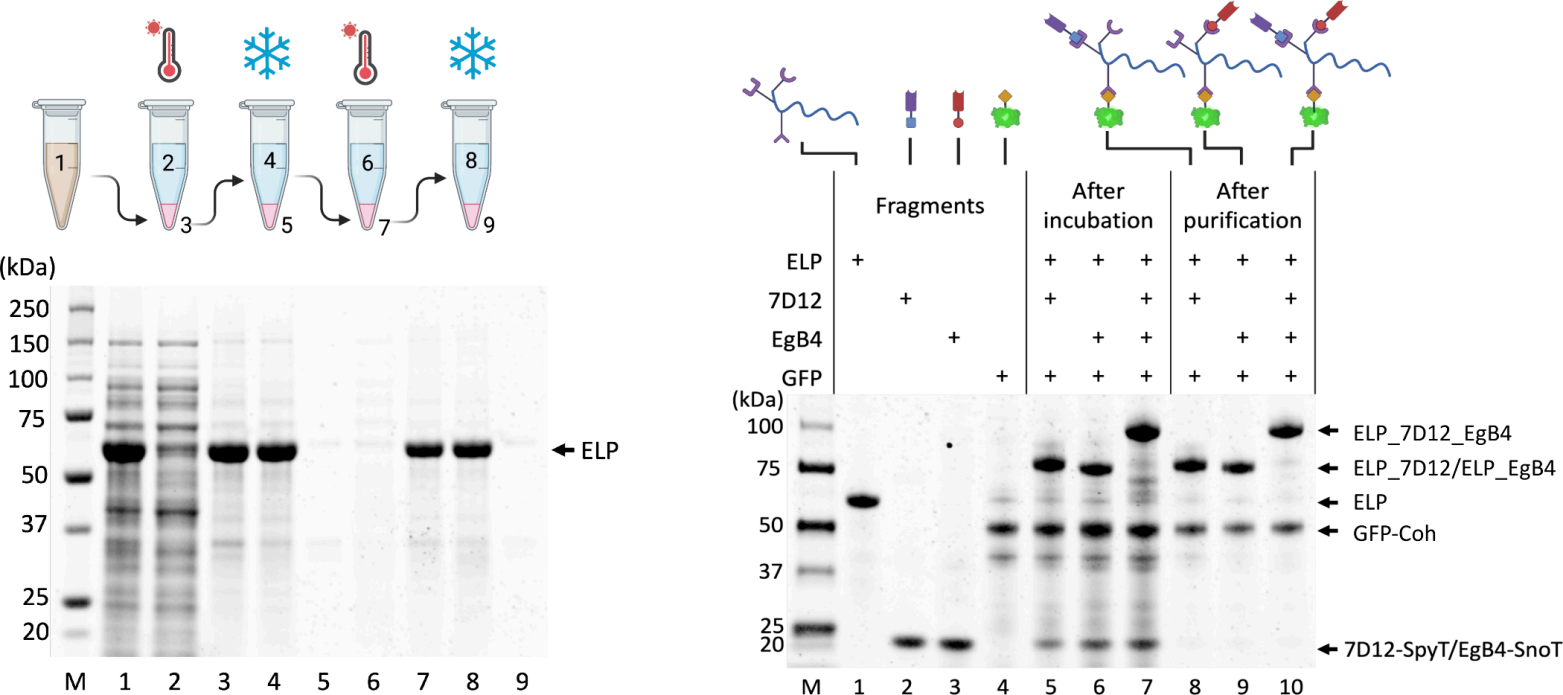
(A) Purification of the ELP scaffold (55.7 kDa) using 1 M (NH_4_)_2_SO_4_ through two cycles of thermal precipitation and cold resolubilization. Lane 1, total cell lysate; lane 2, supernatant after the first thermal precipitation; lane 3, resolubilized pellet after the first thermal precipitation; lane 4, supernatant containing purified ELP scaffold after the first cold spin; lane 5, resolubilized pellet after the first cold spin; lane 6, supernatant after the second thermal precipitation; lane 7, resolubilized pellet after the second thermal precipitation; lane 8, final purified ELP scaffold product after the second cold spin; lane 9: resolubilized pellet after the second cold spin. (B) Assembly and purification of the antibodies. Lane 1, purified ELP scaffold; lane 2, purified 7D12-SpyT (20.4 kDa); lane 3, purified EgB4-SnoT (20.4 kDa); lane 4, purified GFP-coh (48.7 kDa); lanes 5–7, after incubating 7D12-SpyT, EgB4-SnoT, and both 7D12 and EgB4 with ELP scaffold and GFP-coh; and lanes 8–10, purified assembled antibody with 7D12 only (76.1 kDa), EgB4 only (76.1 kDa), and with both 7D12 and EgB4 (96.5 kDa).

The ELP-SpyCather002-SnoopCather-Dockerin scaffold was expressed in BL21(DE3) and purified via two cycles of thermal precipitation with 1 M (NH_4_)_2_SO_4_ and resolubilization in ice-cold PBS (Figure 2A).^29^ We first assembled the antibody with 7D12 and EgB4, which target different regions of EGFR.^30^ As such, an assembled antibody integrating both 7D12 and EgB4 is expected to achieve a greater binding efficiency than a single nanobody in isolation. Nanobodies 7D12 and EgB4, fused with SpyTag002 and SnoopTag, respectively, were expressed in *E*. *coli* SHuffle T7, ensuring correct disulfide bond formation.^31^ Nanobodies fused with SpyTag002/SnoopTag were purified by Strep-Tactin columns through the C-terminal Twin-Strep-Tags. We used GFP as a fluorescent reporter. GFP-cohesin fusion (GFP-coh) was expressed in BL21(DE3). GFP-coh was purified by nickel-nitriolotriacetic acid (Ni-NTA) columns through the C-terminal His-tag.

For antibody assembly, an excess (1.5:1 molar ratio) of nanobody fusions and GFP-coh were incubated with the ELP scaffold. After incubation, the assembled antibody was purified by one round of thermal precipitation and cold resolubilization to eliminate unbound nanobodies and GFP-coh. The purified antibody was analyzed by SDS-PAGE (Figure 2B). Over 90% of the ELP scaffold was assembled with the nanobodies and GFP based on the SDS image analysis. Since GFP-coh was assemble onto the ELP scaffold through cohesin–dockerin noncovalent interaction, GFP-coh appeared on the SDS gel as a separate band. Furthermore, the assembled antibodies were recovered after one cycle of thermal precipitation and cold resolubilization resulting in >98% purity (Figure 2B). Since cohesin–dockerin interaction is noncovalent, GFP-coh appeared as a separate band on SDS-PAGE (Figure 2B).

### Cancer Detection

**Figure 3:**
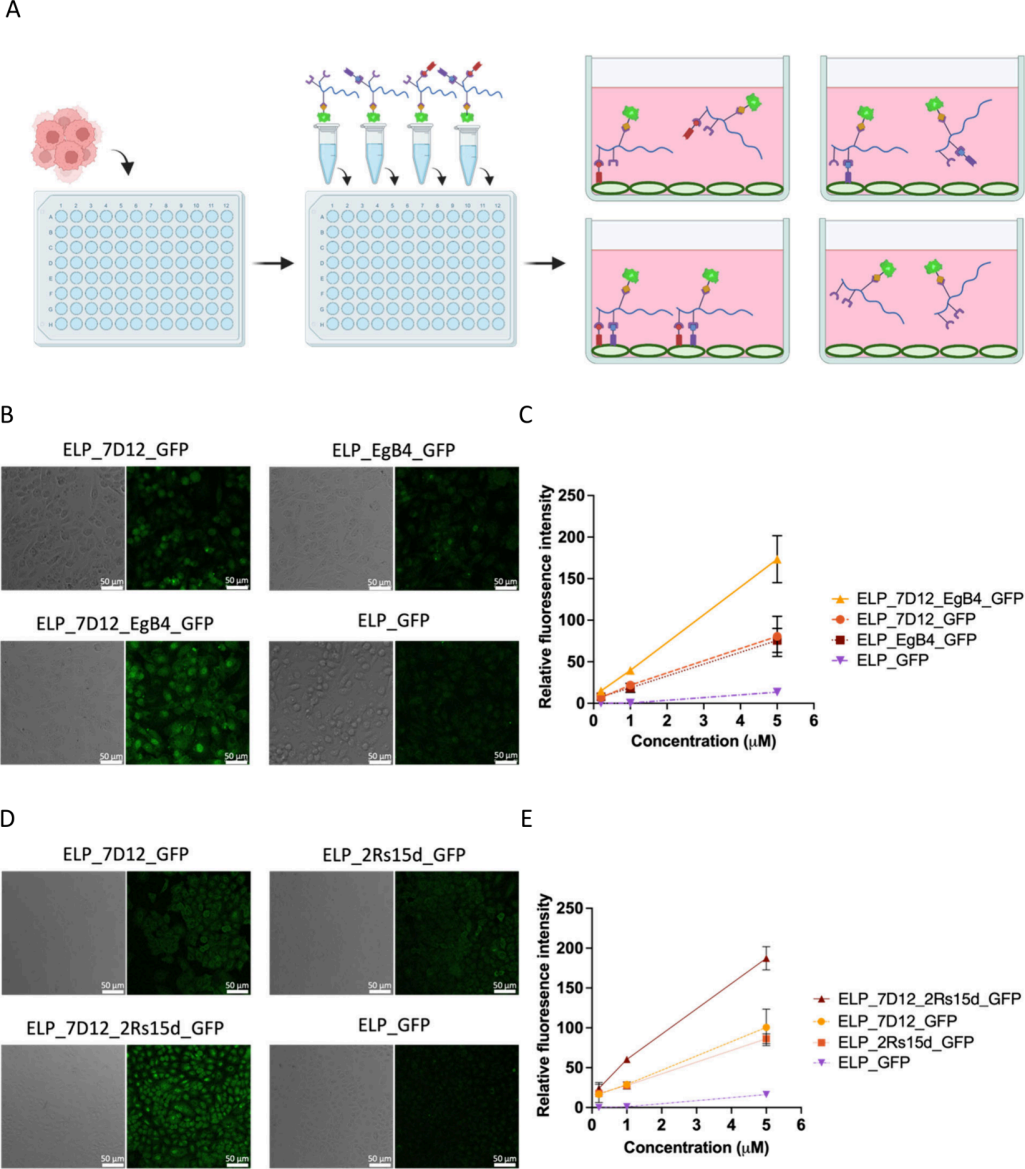
(A) Illustration of the antibody binding assay. Phase contrast and fluorescence images of (B) MDA-MB-231 and (D) A549 cells incubated with 5 μM different antibodies using GFP as the detection output. Fluorescence intensity quantification of (C) MDA-MB-231 and (E) A549 cells incubated with different antibodies. (For C and E, triplicates were tested for each condition; *n* = 3, mean ± SD.)

After antibody assembly, we tested cancer detection using MDA-MB-231, an epithelial human breast cancer cell line that overexpresses EGFR. We hypothesized that multivalence binding using our multifunctional antibody would enable a higher binding efficiency (Figure 3A). The assembled antibody with single nanobody 7D12 or EgB4, which binds onto different regions of EGFR, and both nanobodies were tested by incubating them with MDA-MB-231. The scaffold with only GFP-coh was included as a negative control. As shown from the image results, fluorescence intensity from the cells with assembled 7D12 and EgB4 showed two times higher fluorescence than with each nanobody alone. The scaffold without nanobody only showed little fluorescence background (Figure 3B, 3C). To showcase the modularity of this assembly method, we prepared another antibody combination featuring a 7D12 nanobody targeting EGFR and a 2Rs15d nanobody targeting HER2 using the same procedure. The assembled antibody was then incubated with A549 lung carcinoma epithelial cells that overexpressed both EGFR and HER2 antigens. Similarly, the highest fluorescence intensity was observed from the antibody with both 7D12 and 2Rs15d (Figure 3D, 3E). In addition, we also compared the binding efficiency of the assembled antibodies, including 7D12 with EgB4 and 7D12 with 2Rs15d purified by either thermal precipitation or column purification. Both assembled products had similar binding efficiencies using either of the purification methods (Figure S2). Using this assembly method, we showcased efficient, precise, easily purifiable, and modular antibodies with nanobodies and reporter fluorescent protein for cancer cell detection.

### Cancer Cell Growth Inhibition

**Figure 4:**
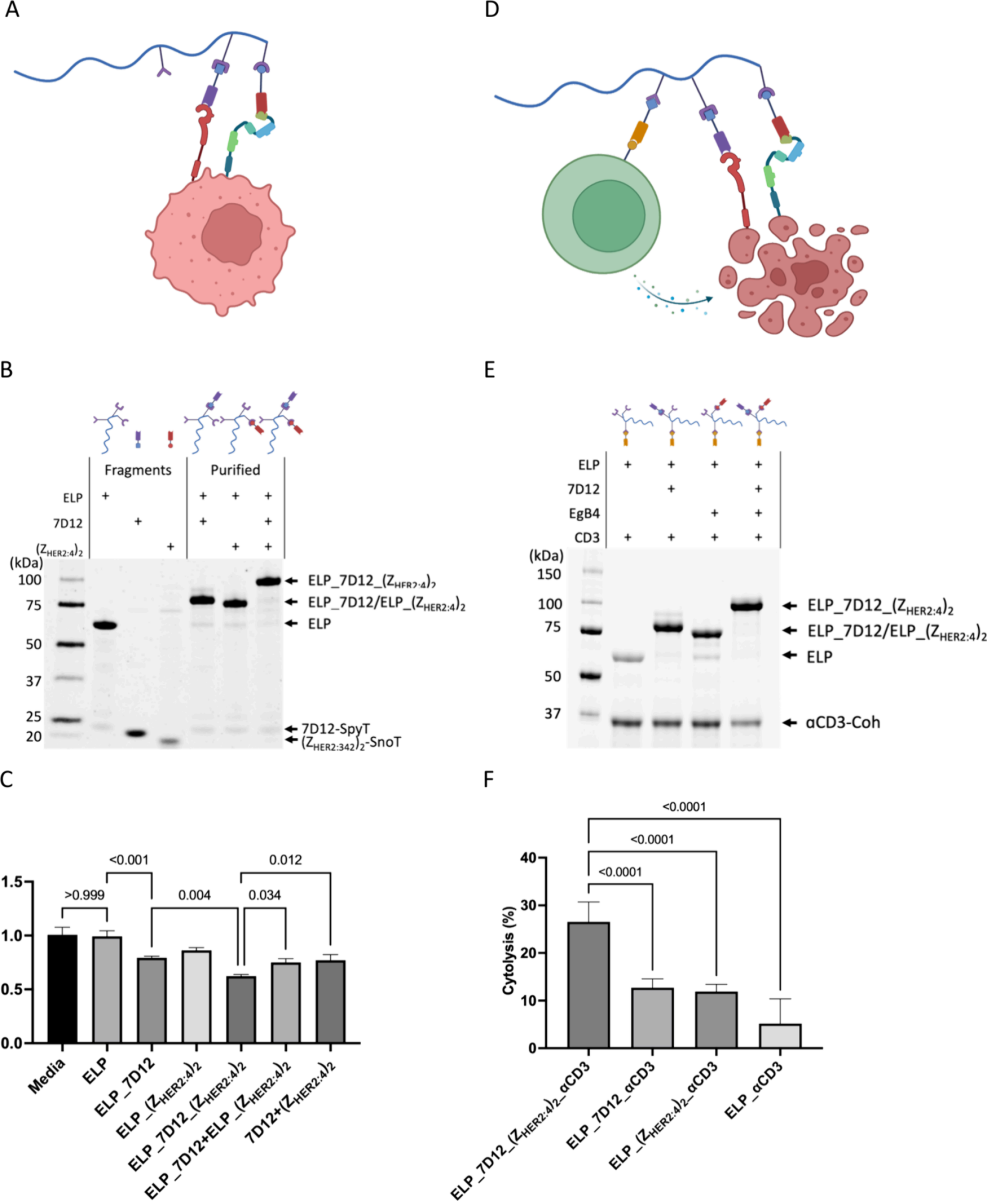
(A) Schematic of using the assembled antibody to inhibit cancer cell growth. (B) SDS of antibody assembly for cancer cell growth inhibition. Lane 1, ELP scaffold (55.7 kDa); lane 2, 7D12-SpyT (20.4 kDa); lane 3: (Z_HER2:4_)_2_-SnoT (19.6 kDa); lane 4: assembled 7D12 (76.1 kDa); lane 5: assembled (Z_HER2:4_)_2_ (75.3 kDa); assembled 7D12+(Z_HER2:4_)_2_ (95.7 kDa). (C) Relative growth inhibition measured by MTT assay at a concentration of 5 μM for each assembled or mixed components including ELP, ELP_7D12, ELP_(Z_HER2:4_)_2_, ELP_7D12_(Z_HER2:4_)_2_, ELP_7D12+ELP_(Z_HER2:4_)_2_, ELP_7D12+ELP_(Z_HER2:4_)_2_, and 7D12+ (Z_HER2:4_)_2_. (D) Schematic of using the assembled antibody to direct T-cells for cancer cell cytolysis. (E) SDS of antibody assembly for T cell mediated cytolysis (lower band on SDS indicates anti-CD3-Coh at 36.8 kDa). (F) T cell mediated cytolysis measured by the LDH assay at a concentration of 1 μM for each assembled antibody. (For C and F: triplicates were tested for each condition, and results were analyzed by one-way ANOVA with Tukey correction for multiple comparisons; *n* = 3, mean ± SD; “–” indicates the assembled components, and “+” indicates mixture of different products.)

Besides detection, we tested the assembled antibodies for their ability to inhibit cancer cell growth (Figure 4A). Nanobody 7D12 has been shown to inhibit cancer cell growth through binding with EGFR.^28,32^ Besides nanobodies, we also utilized affibody (Z_HER2:4_)_2_ that targets HER2 to demonstrate the versatility of this approach.^28,33^ Similar to previous experiments for detection, nanobody 7D12 and affibody (Z_HER2:4_)_2_ were assembled on the protein scaffolds, followed by one round of thermal precipitation and cold resolubilization. SDS-PAGE gel analysis using Image Lab showed that over 90% of the ELP was assembled with over 90% purity (Figure 4B).

Previous studies demonstrated that simultaneously targeting EGFR and HER2 would lead to superior therapeutic efficacy in suppressing tumor growth.^34^ We hypothesized that the assembled antibody targeting both EGFR and HER2 antigens on A549 cells would inhibit cancer cell growth more effectively.^35^ First, the protein scaffold ELP did not show a significant inhibitory effect on A549 cell growth since no significant difference was observed between control (media) and ELP samples (Figure 4C). The cells incubated with 7D12 antibody had a 20% decrease in the overall growth. Meanwhile, (Z_HER2:4_)_2_ alone on the scaffold did not show a significant effect in inhibiting the cell growth, potentially because it was unable to inhibit HER2 phosphorylation.^36^ Excitingly, the assembled antibody ELP_7D12_(Z_HER2:4_)_2_ with both 7D12 and (Z_HER2:4_)_2_ assembled on the protein scaffold showed the most significant growth inhibition for A549 cells (Figure 4C). Compared with the cells grown in media, there was a 37% reduction in cell growth using ELP_7D12_(Z_HER2:4_)_2_. Sample 7D12+(Z_HER2:4_)_2_ (mixture of 7D12+(Z_HER2:4_)_2_) and ELP_7D12+ELP_(Z_HER2:4_)_2_ (mixture of assembled ELP_7D12 and ELP_(Z_HER2:4_)_2_) had only around a 25% reduction in the growth (Figure 4C). Taken together, these results suggest that assembling both 7D12 and (Z_HER2:4_)_2_ onto the ELP scaffold inhibited cell growth most effectively compared with having the nanobody or affibody alone or mixed.

### T Cell Mediated Cytotoxicity

To further improve the therapeutic efficacy and demonstrate the modularity of this method, we added an anti-CD3 nanobody onto the protein scaffold in addition to 7D12 and (Z_HER2:4_)_2_. The effectiveness of anti-CD3 nanobodies in both binding to and activating T-cells has been demonstrated.^37^ This way, the antibody targets EGFR and HER2 simultaneously to inhibit cancer cell growth and recruits and activates T cells to mediate tumor lysis (Figure 4D). Similar to previous experiments, we assembled 7D12, (Z_HER2:4_)_2_, and anti-CD3 nanobodies onto the scaffold and evaluated the assembly by SDS-PAGE. Similar to GFP-coh, anti-CD3 nanobody was assembled through cohesin–dockerin interaction; it appeared as a separate band on the SDS gel. From the SDS-PAGE gel, over 90% of the scaffold was assembled with antibody fragments (Figure 4E). After the assembly, each assembled antibody product was tested for its ability to induce cancer cell cytolysis against A549 cells. At an effect-to-target ratio of 10:1 using mouse CD3+ T cells and A549 cells and a concentration of 1 μM for each assembled antibody, the antibody with 3 nanobodies targeting all CD3, HER2, and EGFR displayed the highest cytolysis (∼27%). In contrast, the antibody with only an anti-CD3 nanobody showed the least cytolysis (Figure 4F). These results confirm that our assembled multifunctional antibody was able to recruit T cells to cancer cells and induce T cell mediated cytolysis against cancer cells. In addition, we also compared the cytolysis effect of the assembled antibody and the nanobody/affibody added in a mixture with or without ELP and observed the highest cytolysis from the assembled antibody (Figure S3). The modularity of our platform will enable more possible antibody compositions for the flexibility of targeting different tumors and recruiting immune cells more efficiently.

## Conclusions

The demand for rapidly developing multifunctional antibodies is increasing. We have developed a modular, easy-to-purify platform that efficiently assembles multifunctional antibodies for cancer detection and therapy. This scaffold, featuring sequentially arranged SpyCatcher002, SnoopCatcher, and Dockerin with (G_4_S)_3_ flexible linkers, enables precise alignment of antibody fragments without steric hindrance (Figure S1). If needed, longer linkers can be incorporated for increased spatial flexibility.

Our highly specific assembly approach allows for efficient and cost-effective integration of antibody fragments, reporters, and immune cell engagers. Expanding this system with additional protein-binding modules, such as SdyTag/SdyCatcher, could further enhance antibody diversity.^38^ ELP simplifies purification while potentially extending antibody half-life.^39^ Using this method, we successfully assembled nanobodies, affibodies, and fluorescent proteins for cancer detection, tumor growth inhibition, and T-cell recruitment.

While our platform improves antibody assembly and therapeutic efficacy, limitations exist due to the smaller size and lower binding affinity of nanobodies and affibodies compared to full-size antibodies.^40^ Incorporating strong binding modules, such as protein A for IgG assembly, could further enhance the functionality. Additionally, future adaptations could enable simultaneous engagement of multiple T-cell recruitment and activation modules for improved therapeutic outcomes.

Our *in vitro* results validate the feasibility of this platform. Moving forward, *in vivo* studies will further investigate its mechanism and therapeutic potential. With its modularity, ease of purification, and spatial control, our platform addresses existing challenges, offering a promising strategy for enhancing antibody-based cancer therapy.

## Methods

### Cloning and Expression

Nanobody and affibody gene sequences were ordered from Twist Bioscience and codons optimized for *E. coli* expression. The ELP scaffold was constructed by first ligating Dockerin, SpyCatcher002, and SnoopCather gene fragments using Gibson assembly with (G_4_S)_3_ in between. The Gibson assembly product was then inserted into the pET24a-ELP-LPEGT plasmid between *Bam*HI and *Xho*I (Figure S4(A), Figure S5(A)).^41^ For each nanobody and affibody, their sequences with a following G_4_S linker were inserted into pET21a after RBS between *Xba*I and *Bam*HI. Protein interaction pairs (Coh, SpyT, SnoT) with another G4S both upstream and downstream the sequence were then inserted after the nanobodies between *Bam*HI and *Eco*RI. Another G_4_S linker and Twin-strep tag were last inserted at the C-terminal between *Eco*RI and *Xho*I (Figure S4(B), Figure S5(B–E)).

The ELP scaffold plasmid was transformed into BL21(DE3) for protein expression. Transformed bacteria were grown in Luria–Bertani (LB) medium supplemented with 50 μg/mL kanamycin, 1.5% glycerol, and 20 mM calcium chloride for proper dockerin folding. The culture was kept at 37 °C until the OD_600_ reached 0.6 and then induced with 0.4 mM IPTG. The induced culture was kept at 30 °C overnight for protein expression. For the nanobodies and affibody expression, each plasmid was transformed into Shuffle T7 for proper disulfide bond formation. The bacteria were cultured in Terrific Broth (TB) at 37 °C until the OD_600_ reached 0.6 and then induced with 0.4 mM IPTG. After induction, the cultures were moved to 30 °C overnight for protein expression.

### Protein Purification

The ELP scaffold was purified by thermal precipitation and cold resolubilization. After the overnight culture for protein expression, the cells were harvested and sonicated to obtain a cell lysate. The cell lysate was then added with (NH_4_)_2_SO_4_ to a final concentration of 1 M, then incubated at 37 °C until the lysate turned cloudy. The lysate was then centrifuged at 37 °C and 20000*g* for 15 min. Supernatant was removed to remove the soluble impurities, and the pellet was resolubilized in cold PBS. After resolubilization, the solution was again centrifuged at 4 °C and 20000*g* for 15 min. Supernatant was transferred to a new tube, and the pellet that contained insoluble impurities was removed. This cycle was repeated to further improve the purity of the final product.

For the nanobodies and affibody, since they contain a Twin-strep tag at the C-terminal, they were purified through the Strep-Tactin column (IBA-Lifesciences) following the manufacturer’s instruction. Briefly, the column was first equilibrated with 2 column volumes (CV) of wash buffer (100 mM pH 8.0 Tris/HCl, 150 mM NaCl, and 1 mM EDTA). Then the cell lysate was loaded onto the column. After that, the column was washed with 5 CV of wash buffer to remove nonspecifically bound proteins. Then the protein was eluted by 3 CV of elution buffer (100 mM Tris/HCl (pH 8.0), 150 mM NaCl, 1 mM EDTA, 50 mM biotin). The column was then regenerated by 6 CV of regeneration buffer (3 M MgCl_2_) followed by equilibration again with 8 CV of wash buffer.

### Antibody Assembly

Multifunctional antibodies were assembled by saturating the ELP scaffold to ensure complete binding of antibody fragments. Prior to assembly, the concentrations of antibody fragments and the ELP scaffold were quantified by using a Nanodrop spectrophotometer. For the assembly, nanobodies or affibodies were mixed with the ELP scaffold at a molar ratio of 1.5:1. When additional fragments were required, all components were mixed together in a one-pot mixing step with each antibody fragment combined with the ELP scaffold at a consistent 1.5:1 molar ratio. The mixture was incubated on a shaking platform at room temperature (60 rpm) for 4 h. After incubation, a 2 μL sample was collected for SDS analysis to monitor the assembly progress.

Following incubation, the assembled antibodies underwent an additional purification step, involving one round of thermal precipitation followed by cold resolubilization to remove any unbound nanobodies or affibodies. Purified products were then analyzed by SDS to confirm the removal of free antibody fragments. Gel images were analyzed by using Bio-Rad Image Lab software. Assembly efficiency was calculated as the ratio of the free ELP band intensity remaining in the final product to the total intensity in that lane. Product purity is calculated as the final product band intensity (including the GFP-coh or αCD3-coh band) over the total lane intensity. Assembled products were stored at −20 °C until use.

### Cancer Cell Detection

MDA-MB-231 (EGFR^+^) and A549 (HER2^+^, EGFR^+^) cells were obtained from the American Type Culture Collection (ATCC). MDA-MB-231 cells were cultured at 37 °C in a non-CO_2_ humidified incubator with Leibovitz’s L-15 medium containing 10 vol % FBS and 1 vol % Pen/Strep. A549 cells were cultured at 37 °C in a 5% CO_2_ humidified incubator with F12-K medium supplemented with 10 vol % FBS and 1 vol % Pen/Strep.

For the *in vitro* cell surface ligand binding assay, cells were seeded into black-wall, transparent-bottom 96-well plates and cultured at 37 °C until reaching around 90% confluency. Once the cells reached around 90% confluency, the growth media was removed, and cells were washed twice with PBST (1× PBS with 0.05% Tween 20). Cells were then fixed with 100 μL of 4% formaldehyde for 15 min and washed three times with 0.05% PBST. To minimize background adsorption, cells were incubated with 5% BSA for 15 min to block unspecific binding and washed three times with 0.05% PBS.

Assembled antibodies were added to cells in specified concentrations for binding at 25 °C. After 1 h of incubation on a horizontal shaker, cells were washed three times with PBST to remove unbound antibodies before measuring the GFP fluorescence in 0.05% PBST with a plate reader. Cells without antibodies were also measured and recorded as a background. Cells incubated with 5 μM assembled antibodies were also imaged using a Leica DMi8 fluorescence microscope.

### Cancer Cell Growth Inhibition

Therapeutic efficacy of the assembled antibodies was evaluated by a cell growth inhibition assay using MTT (ThermoFisher). A549 cells were seeded in 96-well plates and allowed to grow at 37 °C overnight. Assembled antibodies were then added to the cells at a concentration of 5 μM and were incubated with the cells for an additional 72 h. After the incubation, media in the wells were replaced with 12 mM MTT dissolved in PBS and incubated for another 4 h. MTT crystals formed at the end of the 4 h incubation were dissolved in DMSO, and absorbance was measured at 540 nm using a plate reader. Relative cell growth was calculated as Relative growth = (Abs_sample_ – Abs_10%DMSO_)/(Abs_media_ – Abs_10%DMSO_).

### T Cell Mediated Cytolysis

For T cell mediated cytolysis, A549 cancer cells and mouse CD3+ T cells were mixed in a 10:1 ratio in each well of the 96-well plates. Each of the assembled antibody product was added into the wells at a concentration of 1 μM. After addition of the antibodies, the cells were then incubated at 37 °C in a 5% CO_2_ humidified incubator for 48 h. T cell mediated cytolysis was measured by a lactate dehydrogenase (LDH) assay kit (ThermoFisher) following the manufacturer’s instruction. Spontaneous LDH levels were determined by wells with only A549 cells and T cells but without any antibody treatment. Maximum LDH levels was measured by incubating A549 cells with lysis buffer for 45 min. Cytolysis percentage was then calculated by the following formula: Cytolysis = (Abs_sample_ – Abs_spontaneous_)/(Abs_maximum_ – Abs_spontaneous_) × 100%.

## Supporting Information Available

Schematic illustration of the ELP scaffold with dockerin; GFP fluorescence intensity indication for binding efficiency of the ELP scaffold assembled with antibodies purified through thermal precipitation and His-tag column purification; cancer cell cytolysis measured by LDH assay; illustration of cloning sites for ELP assembled antibodies; amino acid sequences of the ELP scaffold, antibodies, and protein interaction pairs (PDF)
