# Supplementary material for: Modular Platform for Efficient Assembly of Multifunctional Antibodies Using Orthogonal Protein–Protein Interactions
Source: ACS Appl Mater Interfaces. 2025 Mar 31;17(14):20685–92. doi: 10.1021/acsami.4c21958 (PMC11986891; doi:10.1021/acsami.4c21958)
Supplement: Supplementary file 1 — am4c21958_si_001.pdf [file am4c21958_si_001.pdf]

Supporting Information

Modular Platform for Efficient Assembly of Multifunctional Antibodies Using Orthogonal Protein-Protein Interactions

Baizhen Gao[1], Rushant Sabnis[1], Siddhi Kotnis[1], Sofia Feliciano[1], Kyge Poling[1], Tracy Mei[1], Min Feng[1], Jugal Kishore Das[2], Jianxun Song[2], Qing Sun[1,3]*

[1] Department of Chemical Engineering, Texas A&M University, College Station, Texas 77840,

United States

[2] Department of Microbial Pathogenesis and Immunology, Texas A&M University Health Science Center, Bryan, TX 77807, United States

[3] Interdisciplinary Graduate Program in Genetics and Genomics, Texas A&M University, College Station, TX, 77843

* Corresponding Author: Qing Sun sunqing@tamu.edu

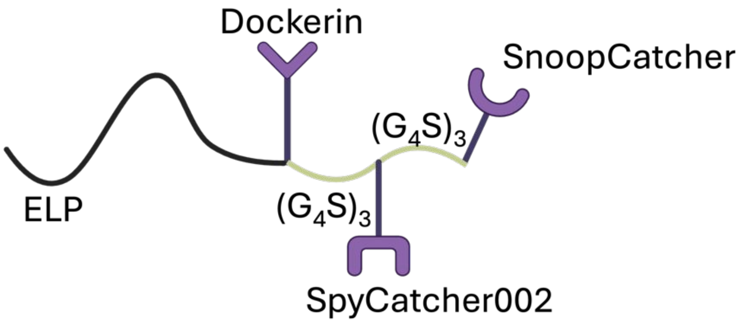

Figure S1. Schematic illustration of ELP scaffold with Dockerin, SpyCatcher002, and SnoopCatcher linked with 2 $(G_4S)_3$ linkers.

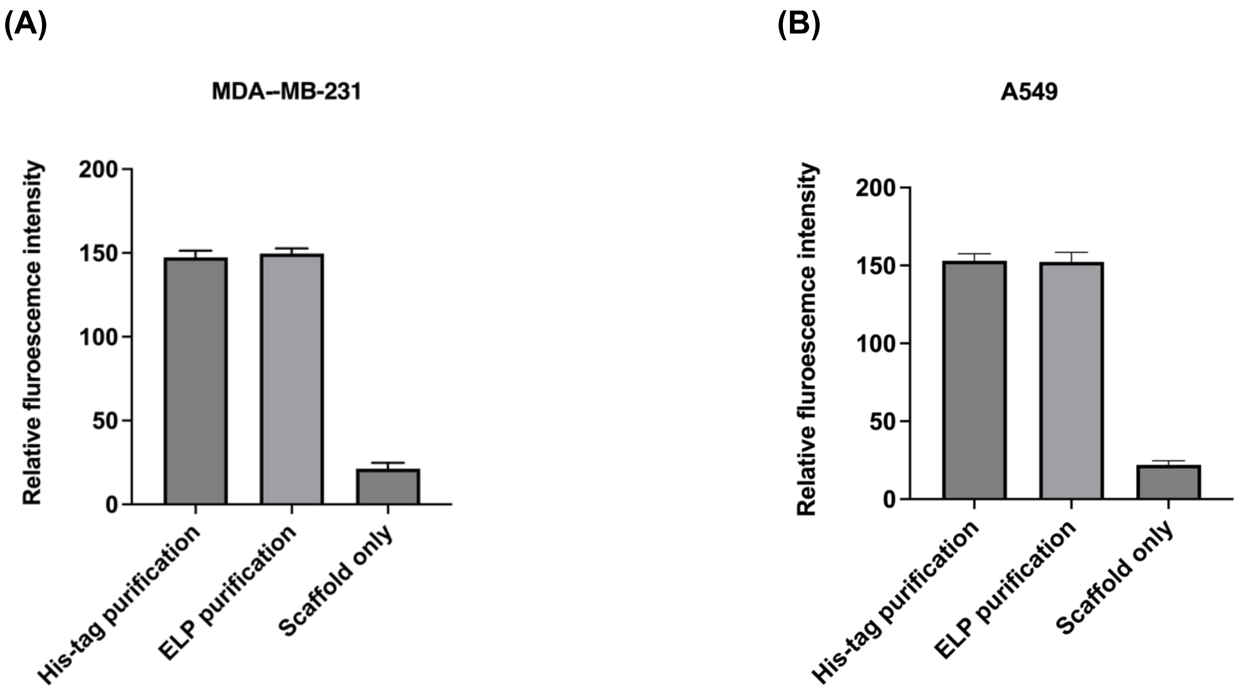

Figure S2. Fluorescence intensity quantification of **(A)** MDA-MB-231 cells incubated with ELP scaffold assembled with 5 μM of 7D12 and EgB4 and ELP scaffold itself and **(B)** A549 cells incubated with ELP scaffold assembled with 5 μM of 7D12 and 2Rs15d and ELP scaffold itself.

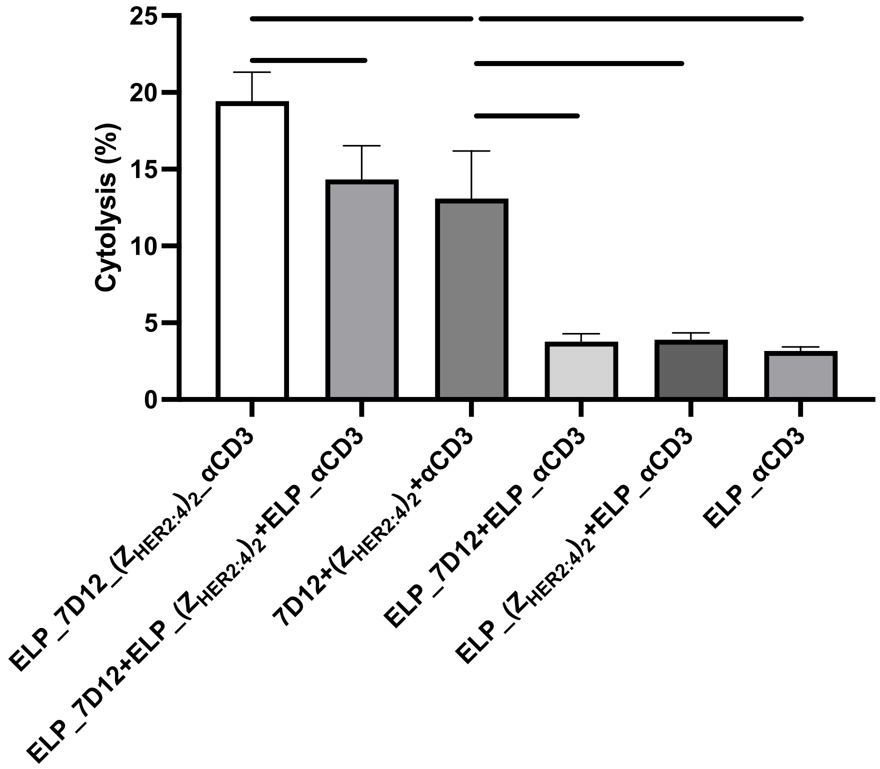

Figure S3. Cytolysis measured by LDH assay at a concentration of 1 μM for each assembled antibody. (n=3, mean±SD, * indicates $p<0.05$, and ** indicates $p<0.01$)

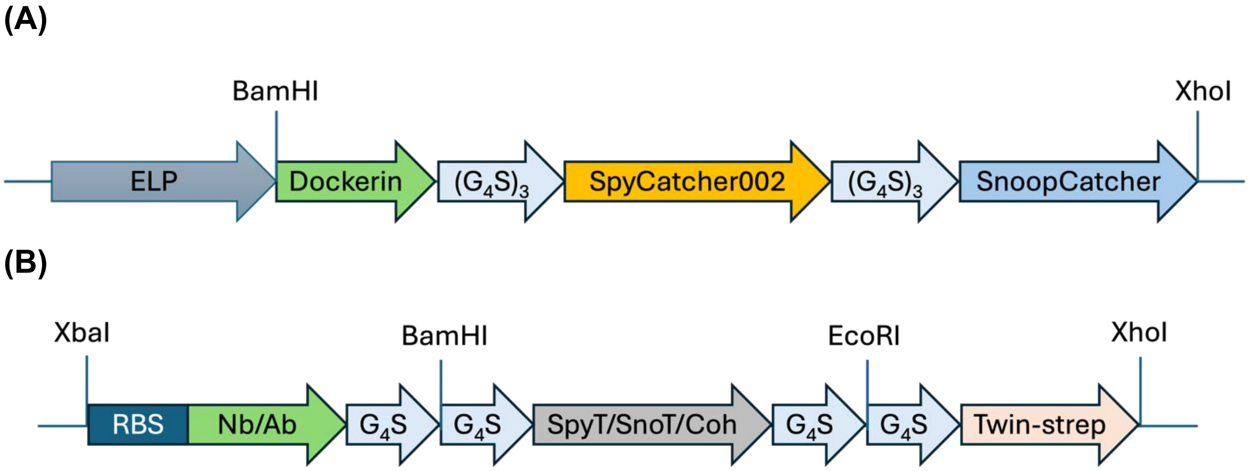

Figure S4. Illustration of the cloning sites for **(A)** ELP scaffold and **(B)** nanobody or affibody

(A)

ELP

SKGPGVGVPGKGVPGVGVPGVGVPGVGVPGVGVPGFGVPGVGVPGVGVPGVGVPGVGVPGKGV

PGVGVPGVGVPGVGVPGVGVPGFGVPGVGVPGVGVPGVGVPGVGVPGKGVPGVGVPGVGVPGV

GVPGVGVPGFGVPGVGVPGVGVPGVGVPGVGVPGKGVPGVGVPGVGVPGVGVPGVGVPGFGVP

BamHI **Dockerin**

GVGVPGVGVPGVGVPGWP<u>GS</u>PRFPNPLSDLSGQPTPPSNPTPSLPPQVVYGDVNGDGNVNSTDL

TMLKRYLLKSVTNINREAADVNRDGAINSSDMTILKRYLIKSIPHLPYGGGGSGGGGSGGGGSMVTT

SpyCatcher002

LSGLSGEQGPSGDMTTEEDSATHIKFSKRDEDGRELAGATMELRDSSGKTISTWISDGHVKDFYLYP

GKYTFVETAAPDGYEVATAITFTVNEQGQVTVNGEATKGDAHTGGGGSGGGGSGGGGSKPLRGAV

SnoopCatcher

FSLQKQHPDYPDIYGAIDQNGTYQNVRTGEDGKLTFKNLSDGKYRLFENSEPAGYKPVQNKPIVAFQ

XhoI

IVNGEVRDVTSIVPQDIPATYEFTNGKHYITNEPIPPKGSG<u>LE</u>HHHHHH

(B)

7D12

QVKLEESGGGSVQTGGSLRLTCAASGRTSRSYGMGWFRQAPGKEREFVSGISWRGDSTGYADSVK

GRFTISRDNAKNTVDLQMNSLKPEDTAIYYCAAAAGSAWYGTLYEYDYWGQGTQVTVSSALEGGGG

SpyTag002 **Twin-strep tag**

SGSGGGGSVPTIVMVDAYKRYKGGGGSEFGGGGSWSHPQFEKGGGSGGGSGGSSAWSHPQFEK

(C)

EgB4

QVQLQESGGGSVQAGGSLKLSCAASGRSFSTYAMGWFRQAPGQDREFVATISWTDSTDYADSVKG

RFTISRDNAKNTGYLQMNSLKPEDTAVYYCAADRWASSRRNVDYDYWGQGTQVTVSSHGSGLVPR

SnoopTag **Twin-strep tag**

GGGGSGSGGGGSGKLGDIEFIKVNKGYGGGGSEFGGGGSWSHPQFEKGGGSGGGSGGSSAWS

HPQFEK

(D)

$(Z_{HER2:4})_2$

VDNKFNKEMRNAYWEIALLPNLNNQQKRAFIRSLYDDPSQSANLLAEAKKLNDAQAPKGGGGSVD

NKFNKEMRNAYWEIALLPNLNNQQKRAFIRSLYDDPSQSANLLAEAKKLNDAQAPKGGGGSGSGG

SnoopTag Twin-strep tag

GGSGKLGDIEFIKVNKGYGGGGSEFGGGGSWSHPQFEKGGGSGGGSGGSSAWSHPQFEK

(E)

Anti-CD3 nanobody

MEVQLVESGGGLVQPGGSLRLSCAASGFTFDDYGMSWVRQAPGKWLEWVSDISWNGGSTYYADS

VKGRFTISRDNAENTLYLQMNSLKPDDTAVYYCAKMGEGGWGANDYWGQGTQVTVSSGGGGSGS

Cohesin

GGGGSPSTQPVTTPPATTKPPATTKPPATTIPPSDDPNAIKIKVDTVNAKPGDTVNIPVRFSGIPSKGIAN

CDFVYSYDPNVLEIIEIKPGELIVDPNPDKSFDTAVYPDRKIIVFLFAEDSGTGAYAITKDGVFATIVAKVK

SGAPNGLSVIKFVEVGGFANNDLVEQRTQFFDGGVNVGDIGGGGSEFGGGGSWSHPQFEKGGGS

Twin-strep tag

GGGSGGSSAWSHPQFEK

Figure S5. Amino acid sequences of **(A)** ELP scaffold, **(B)** 7D12-SpyT, **(C)** EgB4-SnoT, **(D)** $(Z_{HER2:4})_2$-SnoT, and **(E)** anti-CD3-Coh.
